# Emerging biological therapies for psoriatic arthritis: A systematic review

**DOI:** 10.1097/MD.0000000000045259

**Published:** 2025-10-17

**Authors:** Saman Firdous, Hamza Amjad, Muhammad Umair Choudhary, Muhammad Waqas Afzal, Rana Hamza Tayyab, Abida Parveen

**Affiliations:** a Department of Medicine, Ibn e Seena Hospital, Kabul, Afghanistan.

**Keywords:** biologic therapies, DMARDs, efficacy, emerging treatments, IL-17 inhibitors, IL-23 inhibitors, psoriatic arthritis, safety, targeted therapy

## Abstract

**Background:**

Psoriatic arthritis (PsA) is a chronic inflammatory condition characterized by joint involvement, enthesitis, dactylitis, and skin psoriasis. The pathophysiology of PsA is complex, driven by dysregulated immune responses, making targeted therapies essential for managing symptoms. Despite the success of biologic disease-modifying anti-rheumatic drugs (bDMARDs) like tumor necrosis factor (TNF) inhibitors, many patients experience suboptimal responses or adverse effects, necessitating the development of new therapies. Emerging biologics targeting interleukin (IL)-17, IL-23, and Janus kinase (JAK) pathways offer promising alternatives. This review aims to evaluate the efficacy and safety of emerging biologics for PsA.

**Methods:**

A systematic review of studies published from 2000 to December 2024 was conducted. Randomized controlled trials (RCTs), cohort studies, and clinical trials were included to assess the efficacy, safety, and adverse effects of bDMARDs and targeted synthetic DMARDs (tsDMARDs) for PsA treatment. Studies focusing on other aspects of psoriasis or nonpharmacological treatments were excluded. Key data extracted included study design, treatment type, efficacy measures (e.g., ACR response), and safety outcomes.

**Results:**

A total of 20 studies involving 77,124 participants were reviewed. Biologic DMARDs such as TNF inhibitors, IL-17 inhibitors (e.g., secukinumab, ixekizumab), and IL-23 inhibitors (e.g., guselkumab) showed high efficacy in managing both joint and skin symptoms. Emerging therapies like bimekizumab (targeting IL-17A and IL-17F) demonstrated enhanced efficacy compared to traditional biologics. Conventional DMARDs, such as cyclosporine and leflunomide, were less effective but still showed moderate benefits. Safety profiles were generally favorable, with biologics showing fewer adverse effects than conventional therapies.

**Conclusion:**

Emerging biologics have significantly advanced the management of PsA, offering greater efficacy and safety compared to conventional DMARDs. IL-17 and IL-23 inhibitors, along with newer agents like bimekizumab, present promising treatment options for patients with inadequate responses to TNF inhibitors. Personalized treatment strategies, based on disease phenotype and individual patient needs, are essential for optimizing outcomes in PsA management. Further research into long-term efficacy and safety is required to refine treatment protocols and improve patient outcomes.

## 1. Introduction

Psoriatic arthritis (PsA) is a chronic, immune-mediated inflammatory disease characterized by joint inflammation, enthesitis, dactylitis, and skin and nail psoriasis.^[1]^ It significantly impacts patients’ quality of life, leading to pain, joint damage, and functional disability.^[2]^ The pathogenesis of PsA is complex, involving dysregulated immune responses mediated by pro-inflammatory cytokines such as tumor necrosis factor-alpha (TNF-α), interleukin-17 (IL-17), and interleukin-12/23 (IL-12/23), along with various other inflammatory mediators and immune cell pathways.^[3]^ These insights into disease mechanisms have driven the development of targeted biologic therapies designed to interrupt these immune pathways and reduce inflammation.

Biologic disease-modifying anti-rheumatic drugs (bDMARDs) have transformed PsA management over the past 2 decades.^[4]^ The earliest biologics introduced were TNF inhibitors (e.g., etanercept, infliximab, adalimumab), which work by blocking TNF-α, a central cytokine in the inflammatory cascade of PsA. Following this, IL-12/23 inhibitors such as ustekinumab were developed to target the p40 subunit common to both IL-12 and IL-23, thereby modulating Th1 and Th17 cell differentiation. More recently, IL-17 inhibitors (e.g., secukinumab, ixekizumab) have gained prominence by directly targeting IL-17A, a key cytokine in PsA pathophysiology responsible for neutrophil recruitment and tissue inflammation.^[5]^ Additionally, small molecule inhibitors such as Janus kinase (JAK) inhibitors (e.g., tofacitinib, upadacitinib) disrupt intracellular signaling of multiple cytokines, further expanding therapeutic options.

Despite these advances, a considerable proportion of patients experience suboptimal or diminishing responses, adverse effects, or intolerance to current biologics, underscoring the need for newer and more refined therapeutic strategies.^[6]^

Emerging biological therapies are now being developed to address these limitations. These include novel agents that selectively target IL-23 (e.g., guselkumab, risankizumab) without affecting IL-12, or dual inhibitors with broader immunomodulatory effects. These therapies offer alternative mechanisms of action, potential improvements in efficacy, and possibly more favorable safety profiles.^[7]^

This systematic review aims to evaluate the current landscape of emerging biologics for PsA, with a focus on their mechanisms of action, clinical efficacy, safety, and potential role in routine clinical practice. Applying the PICO (Population, Intervention, Comparator, Outcome) framework, this review will examine adult patients with psoriatic arthritis (Population), treated with novel or emerging biologic therapies (Intervention), compared to placebo or established biologics such as TNF, IL-17, or IL-12/23 inhibitors (Comparator), assessing outcomes related to disease activity, quality of life, functional status, and adverse events (Outcome). Particular attention will be given to characterizing the study populations across all included trials, including baseline demographics, disease severity, treatment history, and comorbidities, to provide a nuanced understanding of treatment responses and generalizability of findings.

## 2. Methods

**Figure 1.**
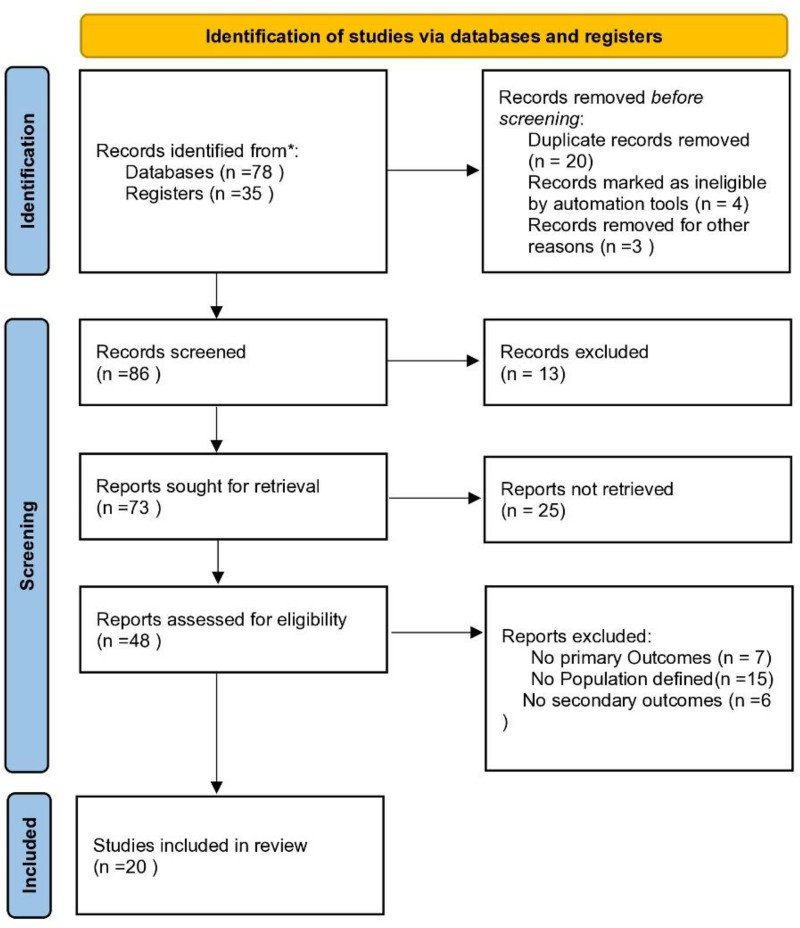
Prisma flowchart.

This systematic review aimed to evaluate the efficacy and safety of various disease-modifying anti-rheumatic drugs (DMARDs) in the treatment of psoriatic arthritis (PsA). The review included original studies published up to the year 2024, with a specific focus on well-defined study designs such as randomized controlled trials (RCTs), prospective and retrospective cohort studies, and interventional clinical trials directly assessing pharmacological treatments for PsA. Case series, case reports, editorials, and nonclinical or preclinical studies were excluded. The review was conducted in accordance with the PRISMA (Preferred Reporting Items for Systematic Reviews and Meta-Analyses) 2020 guidelines (Fig. 1). Studies were only included if they primarily addressed therapeutic interventions for PsA, with clearly described patient populations and outcomes relevant to efficacy or safety. Research articles focused on nonpharmacological treatments, surveys, or conditions unrelated to PsA were excluded to ensure methodological consistency and clinical relevance.

### 2.1. Eligibility criteria

The inclusion criteria were studies that specifically evaluated DMARDs, including conventional synthetic DMARDs (csDMARDs), biologic DMARDs (bDMARDs), and targeted synthetic DMARDs (tsDMARDs) in adult patients diagnosed with PsA. Only studies reporting on the efficacy, safety, and adverse effects of these treatments were considered. Studies published in English were included. Observational studies, case reports, and studies not involving pharmacological treatment were excluded to ensure a focused analysis on the therapeutic outcomes for PsA.

### 2.2. Data sources and search strategy

A comprehensive literature search was conducted in major electronic databases including PubMed, Cochrane Library, Scopus, and Web of Science. The search covered studies published from January 2000 to December 2024. A combination of Medical Subject Headings (MeSH) terms and free-text keywords was used, including: *“psoriatic arthritis,” “PsA,” “disease-modifying antirheumatic drugs,” “DMARDs,” “conventional DMARDs,” “biologic therapy,” “biologic DMARDs,” “targeted synthetic DMARDs,” “JAK inhibitors,” “IL-17 inhibitors,” “IL-23 inhibitors,” and “TNF inhibitors.”* Boolean operators (AND, OR) were applied to combine search terms. Reference lists of relevant articles and reviews were also screened to identify additional eligible studies.

### 2.3. Study selection and data extraction

Studies were screened for relevance based on title and abstract, followed by full-text review to confirm eligibility. Key data extracted from the included studies included the study design, total number of participants, age and gender distribution, type of treatment, primary and secondary outcomes, adverse effects, and treatment efficacy as measured by established clinical response criteria (e.g., ACR response, psoriatic arthritis response criteria [PsARC], ACR20, ACR50, and ACR70).

### 2.4. Quality assessment

The quality of the studies included was assessed using standard tools such as the Cochrane Risk of Bias Tool for RCTs and the Newcastle-Ottawa Scale for cohort studies. Studies with high risk of bias or methodological limitations were critically analyzed, and those deemed to have insufficient quality were excluded from the final synthesis.

### 2.5. Data synthesis

Data from the included studies were synthesized qualitatively. The primary focus was on comparing the efficacy of different DMARDs in reducing PsA symptoms, including joint inflammation and skin lesions, as well as the safety profile, highlighting adverse effects. Results were summarized narratively, and key findings were presented in a tabular format for clarity.

## 3. Results

A total of 20 studies involving 77,124 participants were included in this systematic review. These studies assessed the efficacy and safety of various DMARDs for the treatment of PsA, including conventional synthetic DMARDs (csDMARDs), biologic DMARDs (bDMARDs), and targeted synthetic DMARDs (tsDMARDs). The review comprised RCTs, clinical trials, and large-scale cohort studies.

### 3.1. Overview of included studies

Table 1 provides a comprehensive overview of key studies evaluating DMARDs in PsA, summarizing study design, participant characteristics, therapeutic class, mechanisms of action, primary outcomes, and adverse effects. This summary allows direct comparison of clinical effectiveness and tolerability across treatment classes.

**Table 1:** Summary of key studies on the efficacy and safety of DMARDs in psoriatic arthritis.

Year	Author (first author et al)	Study design	Participants (n)	Age (mean ± SD, years)	Gender (n, %)	Therapeutic class	Agent	MOA	Outcomes (primary/secondary)	Treatment-related adverse effects	Efficacy
2019^[8]^	Ribeiro da Silva M.R., et al	Prospective cohort	11,008	NR	NR	bDMARDs	Adalimumab, Etanercept, Infliximab	TNF-α inhibitors	Adalimumab showed higher medication persistence than etanercept or infliximab	NR	Persistence favorable for adalimumab
2000^[9]^	Mease P.J., et al	RCT, double-blind, placebo-controlled	60	NR	NR	bDMARDs	Etanercept	TNF inhibitors	87% met PsARC vs 23% placebo	NR	ACR20: 73% vs 13% placebo
2004^[10]^	Mease P.J., et al	Clinical Trial	205	NR	NR	bDMARDs	Etanercept	TNF inhibitors	Reduced signs and symptoms of PsA	NR	ACR20: 73% vs 15% placebo
2025^[11]^	Möller B., et al	Observational	197	NR	NR	bDMARDs	bDMARD + csDMARD	Various	Retention improved with csDMARD co-therapy	NR	Improved drug survival
2020^[12]^	Mease P.J., et al	Multicenter RCT	1493 (PBO = 495; Apremilast 30mg = 497; Apremilast 20mg = 501)	PBO: 50.5 ± 11.6; A30: 50.6 ± 11.4; A20: 49.8 ± 11.7	Females: 51%–55%	Targeted synthetic DMARDs	Apremilast	PDE4 inhibitor	Long-term treatment safe/tolerated	Diarrhea, nausea, headache, URTI, nasopharyngitis	Sustained efficacy
2008^[13]^	Antoni C.E., et al	RCT	104	NR	NR	bDMARDs	Infliximab	TNF inhibitors	Improved joints, skin, inhibited radiographic progression	NR	ACR20: 62%, ACR50: 45%, ACR70: 35%
2022^[14]^	Deodhar A.A., et al	Clinical Trial	1401	49.1 ± 11.9	M: 679 (48.5%), F: 722 (51.5%)	bDMARDs	Ixekizumab	IL-17A inhibitor	Consistent safety and tolerability	Nasopharyngitis, URTI, bronchitis, sinusitis	Effective
2019^[15]^	Ghosh S., et al	Clinical Trial	1018	47.6 ± 11.9	M: 560 (52.2%), F: 458 (47.8%)	bDMARDs	Ustekinumab	IL-12/23 inhibitor	Favorable safety profile	NR	Effective
2018^[16]^	Deodhar A., et al	Clinical Trial	149 (Guselkumab = 100, Placebo = 49)	NR	NR	bDMARDs	Guselkumab	IL-23 inhibitor	Improved active PsA symptoms	Infection	ACR20: 58% vs 18% placebo
2014^[17]^	Horneff G., et al	Prospective, multicenter	29 (pediatric PsA)	14.5 ± 2.5	F: 23 (79.3%)	bDMARDs	Etanercept	TNF inhibitors	Effective and well tolerated in pediatric PsA	Abdominal pain, pneumonia, gastroenteritis	JIA ACR30: 93%
2007^[18]^	Papp K.A., et al	Clinical Trial	115	NR	NR	bDMARDs	Efalizumab	LFA-1/ICAM-1 inhibitor	Ineffective but no worsening	NR	ACR20 in 28%
2022^[19]^	Mease P.J., et al	Multicenter RCT	203 (PBO = 66; Deucravacitinib 6mg = 70; 12mg = 67)	49.8 ± 13.5	F: 104 (51.2%)	Targeted synthetic DMARDs	Deucravacitinib	JAK inhibitor	Safe, well tolerated	Nasopharyngitis, URTI, rash, diarrhea	ACR20: 52.9–62.7% vs 31.8% PBO
2004^[20]^	Kaltwasser J.P., et al	Clinical Trial	190 (Leflunomide = 98; Placebo = 92)	Leflunomide: 48.6 ± 10; PBO: 46.9 ± 12	M: ~58%	Conventional DMARDs	Leflunomide	Pyrimidine synthesis inhibitor	Effective in PsA	Diarrhea, ↑ALT	PsARC: 58.9% vs 29.7% PBO
2007^[21]^	Genovese M.C., et al	RCT, placebo-controlled	100 (Adalimumab = 51, PBO = 49)	NR	NR	bDMARDs	Adalimumab	TNF inhibitor	Reduced signs, symptoms, disability	NR	ACR20: 65% vs 57%; PsARC: 51% vs 24%
2001^[22]^	Salvarani C., et al	RCT, open-label	99	NR	NR	Conventional DMARDs	Cyclosporine, Sulfasalazine	Immunosuppressants	CSA more effective than ST/SSZ	Mild reversible renal dysfunction	ACR50/70 higher with CSA
2024^[23]^	Watad A., et al	Retrospective cohort	58,671	NR	NR	bDMARDs	Biologics vs MTX	Anti-IL-12/23, IL-23p19, IL-17, TNFi	PsA incidence lower with biologics vs MTX	NR	Lower incidence rates
2023^[24]^	Ritchlin C.T., et al	RCT, multicenter	852 (PBO = 281; BKZ = 431; ADA = 140)	~49 ± 12	M: ~45% to 50%	bDMARDs	Bimekizumab	IL-17A/F inhibitor	Sustained ACR, PASI responses	Candida infections	Joint, skin, disease activity improved
2023^[25]^	Mease P.J., et al	RCT	206	49.3	M: 51%	bDMARDs	Bimekizumab	IL-17A/F inhibitor	Long-term improvements in pain/fatigue	NR	Sustained efficacy
2022^[26]^	McInnes I.B., et al	Clinical Trial	739 (Guselkumab q4w = 245; q8w = 248; PBO = 246)	NR	NR	bDMARDs	Guselkumab	IL-23p19 inhibitor	Clinical improvements (ACR20/50/70)	NR	ACR20: 68%–76%
2022^[27]^	Coates L.C., et al	RCT, double-blind	285 (Guselkumab = 189; PBO = 96)	49 ± 12	F: 52%	bDMARDs	Guselkumab	IL-23p19 inhibitor	Improved joints, skin, function in TNFi-IR PsA	NR	Effective

ACR20 = American College of Rheumatology preliminary criteria for improvement, bDMARDs = biologic disease-modifying anti-rheumatic drugs, CSA = cyclosporine, DMARDs = disease-modifying anti-rheumatic drugs, ICAM-1 = intercellular adhesion molecule-1, IL = interleukin, LFA-1 = lymphocyte function-associated antigen-1, MOA = mechanism of action, PASI = Psoriasis Area and Severity Index, PDE4 = phosphodiesterase 4, PsA = psoriatic arthritis, PsARC = psoriatic arthritis response criteria, SSZ = sulfasalazine, ST = symptomatic therapy, TNF = tumor necrosis factor.

### 3.2. Conventional synthetic DMARDs

Conventional DMARDs such as cyclosporine and leflunomide demonstrated efficacy in managing PsA symptoms.^[8]^ Cyclosporine, studied in 99 participants, yielded superior ACR50 and ACR70 responses compared to sulfasalazine and symptomatic therapy. Reported adverse events included mild, reversible nephrotoxicity. Leflunomide, assessed in a trial with 190 participants, achieved a 58.9% PsARC response rate. However, gastrointestinal discomfort and elevated liver enzymes were common adverse events.^[9–20]^

### 3.3. Biologic DMARDs

Twelve studies assessed biologic DMARDs in over 12,500 patients. These agents demonstrated consistent clinical efficacy and targeted specific immune pathways. TNF inhibitors, including etanercept, infliximab, and adalimumab, achieved high ACR20 response rates ranging from 62% to 87%, along with significant improvements in both skin and joint symptoms. Etanercept was particularly effective in patients with both joint and cutaneous involvement, reflecting the dual action of TNF-α inhibition.^[9]^

The retention rates of TNF inhibitors and other biologics were comparable in patients with both low and high joint counts, suggesting that disease severity based on joint count alone should not guide treatment decisions. In patients with significant symptoms or disease burden despite a low joint count, bDMARDs remain justified due to inflammation in other domains such as skin, entheses, or dactylitis.^[21]^

Biologic therapies, particularly TNF inhibitors, were also shown to reduce the risk of developing PsA in patients with psoriasis.^[22]^ One study reported a hazard ratio (HR) of 0.46 for biologics compared to methotrexate. Among the biologics, incidence rates per 100 person-years were 4.57 for anti-IL-12/23 or IL-23p19, 4.35 for anti-IL-17, and 2.55 for TNF inhibitors. However, no statistically significant differences were observed between these groups in preventing PsA.^[23]^ Additionally, patients receiving phototherapy had a higher risk of developing PsA compared to those using topical therapy (HR = 1.85), suggesting more severe skin disease as a potential risk factor.

### 3.4. Emerging therapies: IL-17 and IL-23 inhibitors

Newer biologics such as bimekizumab (BKZ), a dual IL-17A and IL-17F inhibitor, demonstrated strong and sustained efficacy. In pivotal trials, BKZ maintained robust ACR20/50/70, PASI75/90/100, and minimal disease activity responses from Week 16 through Week 52. Patients switching to BKZ at Week 16 achieved outcomes similar to those initially randomized to BKZ.^[24,28–33]^ The safety profile was acceptable, with adverse events reported in 79.1% of BKZ patients and 80.7% of those on adalimumab. Notable side effects included Candida infections in 7.7% of BKZ patients. One death occurred but was deemed unrelated to the study drug.

BKZ treatment also resulted in significant reductions in fatigue and pain, leading to long-term improvements in quality of life, physical functioning, and overall disease burden. These sustained benefits enhanced the ability of patients to manage PsA over time.^[25]^

### 3.5. Summary of efficacy outcomes

A detailed comparison of efficacy outcomes is presented in Table 2, incorporating the most frequently used outcome measures. The American College of Rheumatology (ACR) response criteria, including ACR20, ACR50, and ACR70, reflect improvements in tender/swollen joint counts and patient-reported outcomes. The Psoriasis Area and Severity Index (PASI) is used to evaluate skin response, with thresholds at PASI75, PASI90, and PASI100 representing 75%, 90%, and 100% improvement, respectively. The Disease Activity Index for Psoriatic Arthritis (DAPSA), which includes joint counts, pain scores, patient global assessment, and C-reactive protein levels, is increasingly used in newer trials.

**Table 2:** Critical appraisal of included studies and knowledge gaps.

Year (ref)	Author	Study strengths	Study limitations	Knowledge gaps/future directions
2019^[8]^	Michael Ruberson Ribeiro da Silva, et al	Very large prospective cohort (n = 11,008); real-world biologic DMARD persistence data	Observational design; potential bias and confounders	Need randomized controlled comparisons of biologics with long-term outcomes
2000^[9]^	P. J. Mease, et al	First double-blind RCT of etanercept; placebo control	Small sample size (n = 60); limited follow-up	Longer duration studies on safety and joint preservation required
2004^[10]^	Philip J. Mease, et al	Large clinical trial (n = 205); robust efficacy of etanercept	Generalizability limited to trial populations	Comparative studies with newer biologics lacking
2025^[11]^	Burkhard Möller, et al	Modern data on bDMARD retention with csDMARD co-therapy	Limited sample size (n = 197); incomplete methodology	Need further RCTs on co-therapy benefits across populations
2020^[12]^	Philip J. Mease, et al	Large multicenter RCT (n = 1493); good safety monitoring	Adverse events common; subgroup analysis limited	Role of apremilast in biologic-refractory PsA unclear
2008^[13]^	Christian E. Antoni, et al	First infliximab RCT; radiographic progression data	Small sample (n = 104); infusion reactions	More data on structural protection and long-term safety needed
2022^[14]^	Atul A. Deodhar, et al	Large trial (n = 1401); IL-17A targeted therapy efficacy and safety	Risk of infections; no long-term follow-up	Comparative effectiveness studies vs TNF and IL-23 inhibitors required
2019^[15]^	Subrata Ghosh, et al	Large sample (n = 1018); ustekinumab safety well described	Focused on safety rather than detailed efficacy	Comparative head-to-head IL-12/23 vs IL-23 inhibitors warranted
2018^[16]^	Atul Deodhar, et al	Guselkumab efficacy demonstrated in active PsA	Small sample (n = 149); short-term results	Need long-term real-world data and infection risk assessment
2014^[17]^	Gerd Horneff, et al	Pediatric PsA study; prospective interventional data	Very small sample (n = 29); not randomized	Need larger pediatric RCTs with longer follow-up
2007^[18]^	Kim A. Papp, et al	First trial on efalizumab in PsA	Ineffective results; small sample (n = 115)	Not pursued further; highlights need for better molecular targets
2022^[19]^	Philip J. Mease, et al	RCT of JAK inhibitor (deucravacitinib); robust efficacy results	Moderate sample size (n = 203); adverse effects common	Long-term JAK inhibitor safety in PsA requires further trials
2004^[20]^	J. Peter Kaltwasser, et al	Leflunomide RCT; good efficacy in PsA	Moderate sample size (n = 190); liver enzyme elevations	Comparative data vs MTX and biologics missing
2007^[21]^	Mark C. Genovese, et al	Placebo-controlled RCT of adalimumab; robust efficacy	Small trial (n = 100); short duration	Long-term functional and radiographic data needed
2001^[22]^	C. Salvarani, et al	Prospective randomized open trial; compared CSA, SSZ, and ST	Small sample (n = 99); open-label bias	Long-term safety of CSA vs conventional underexplored
2024^[23]^	Abdulla Watad, et al	Huge retrospective cohort (n = 58,671); biologics vs MTX	Retrospective design; residual confounding	Prospective validation of PsA prevention with biologics required
2023^[24]^	Christopher T. Ritchlin, et al	Head-to-head trial (BKZ vs ADA); large sample (n = 852)	High cost; limited ethnic diversity	Cost-effectiveness and real-world comparisons needed
2023^[25]^	Philip J. Mease, et al	Long-term bimekizumab trial; sustained improvement in pain/fatigue	Moderate sample size (n = 206); lacks radiographic outcomes	Long-term comparative efficacy and safety data needed
2022^[26]^	Iain B. McInnes, et al	Large trial (n = 739); multiple guselkumab dosing regimens	Limited subgroup analysis; infection risk	Comparative IL-23 inhibitor studies required
2022^[27]^	Laura C. Coates, et al	RCT in TNFi-IR PsA patients; guselkumab improved joint/skin	Moderate sample (n = 285); limited follow-up	Role of guselkumab in early PsA and MTX-naïve patients needs evaluation

CSA = cyclosporine, DMARDs = disease-modifying anti-rheumatic drugs, IL = interleukin, PsA = psoriatic arthritis, RCTs = randomized controlled trials, SSZ = sulfasalazine, ST = symptomatic therapy, TNF = tumor necrosis factor.

These indices are used throughout the included studies and will allow for a structured comparison of the efficacy profiles of TNF inhibitors, IL-17 inhibitors, IL-12/23 blockers, and emerging biologics such as bimekizumab, risankizumab, and guselkumab. This approach aims to determine the relative effectiveness of each drug class in achieving comprehensive disease control in PsA.

Overall, the reviewed studies provide substantial evidence supporting the efficacy and safety of conventional DMARDs, biologic DMARDs, and targeted synthetic DMARDs in psoriatic arthritis, yet several critical gaps remain. While RCTs demonstrate robust short-term efficacy, many suffer from small sample sizes, limited generalizability, and inadequate long-term follow-up. Large cohort studies contribute valuable real-world insights but are often limited by retrospective designs and residual confounding. Head-to-head trials between different biologic and targeted therapies are scarce, leaving comparative effectiveness largely unaddressed. Additionally, pediatric PsA remains underrepresented, and safety data on newer agents such as JAK and IL-23 inhibitors are still emerging. Future research should focus on long-term outcomes, radiographic progression, cost-effectiveness, and personalized treatment strategies to optimize patient care (Table 2).

Analysis of efficacy outcomes across the included studies demonstrates that biologic DMARDs consistently achieve superior clinical responses compared with placebo or conventional DMARDs. TNF inhibitors such as etanercept, infliximab, and adalimumab demonstrated robust improvements, with ACR20 responses ranging from 62% to 73%, and in some cases significant ACR50 and ACR70 responses, as well as inhibition of radiographic progression. Leflunomide and cyclosporine showed moderate efficacy in older trials but were limited by adverse effects and lower overall response rates compared to biologics. More recent trials evaluating IL-17, IL-12/23, and IL-23 inhibitors have reported sustained improvements in joint and skin outcomes, with guselkumab and bimekizumab demonstrating high ACR20/50/70 response rates and marked improvements in PASI scores. Targeted synthetic agents such as apremilast and deucravacitinib offered modest but clinically meaningful benefits, particularly in biologic-refractory populations, though their long-term comparative efficacy remains uncertain. Notably, disease activity indices such as DAPSA were infrequently reported, limiting cross-trial comparisons. Overall, these findings confirm the superior efficacy of biologic therapies in psoriatic arthritis, while highlighting the need for standardized reporting of ACR, PASI, and DAPSA outcomes across future trials to facilitate robust comparative effectiveness assessments (Table 3).

**Table 3:** Summary of efficacy outcomes reported in included studies.

Year (ref)	Author	Intervention	ACR20 (%)	ACR50 (%)	ACR70 (%)	PASI75/90/100 (%)	DAPSA/other efficacy outcomes
2000^[9]^	Mease et al	Etanercept	73	–	–	–	PsARC achieved in 87% vs 23% placebo
2004^[10]^	Mease et al	Etanercept	73	50	30	PASI not reported	Significant reduction in PsA symptoms
2008^[13]^	Antoni et al	Infliximab	62	45	35	PASI not reported	Inhibited radiographic progression
2007^[21]^	Genovese et al	Adalimumab	65	–	–	–	PsARC response 51% vs 24% placebo
2019^[8]^	Ribeiro da Silva et al	Adalimumab/ Etanercept/ Infliximab	Not reported	Not reported	Not reported	Not reported	Adalimumab showed higher drug persistence
2014^[17]^	Horneff et al	Etanercept (pediatric PsA)	–	–	–	–	JIA ACR30 achieved by 93%
2001^[22]^	Salvarani et al	Cyclosporine vs SSZ vs Symptomatic therapy	–	CSA showed higher ACR50 and ACR70	–	–	CSA significantly superior to SSZ and ST
2004^[20]^	Kaltwasser et al	Leflunomide	–	–	–	–	PsARC response 58.9% vs 29.7% placebo
2018^[16]^	Deodhar et al	Guselkumab	58	–	–	PASI not reported	Significant symptom improvement
2022^[26]^	McInnes et al	Guselkumab (q4w/ q8w)	68–76	48–56	30–36	PASI not reported	Clinical improvements across outcomes
2022^[27]^	Coates et al	Guselkumab (TNFi-IR PsA)	Reported improvement	–	–	–	Improved joint, skin, and function
2019^[15]^	Ghosh et al	Ustekinumab	Not reported	Not reported	Not reported	PASI data favorable	Consistent safety profile
2022^[14]^	Deodhar et al	Ixekizumab	Not reported	Not reported	Not reported	PASI improvement consistent	Safe and well tolerated
2023^[24]^	Ritchlin et al	Bimekizumab vs ADA	Sustained ACR20/50/70	–	–	PASI75/90/100 achieved	MDA responses improved
2023^[25]^	Mease et al	Bimekizumab	Sustained ACR responses	–	–	–	Reduced pain and fatigue
2022^[19]^	Mease et al	Deucravacitinib	52.9–62.7	–	–	–	Higher ACR20 vs placebo
2020^[12]^	Mease et al	Apremilast	Not reported	–	–	–	Safe, well tolerated, symptom improvement
2024^[23]^	Watad et al	Biologics vs MTX	Not reported	Not reported	Not reported	Not reported	Biologics reduced PsA incidence

ACR = American College of Rheumatology, CSA = cyclosporine, DAPSA = Disease Activity Index for Psoriatic Arthritis, MDA = minimal disease activity, PASI = Psoriasis Area and Severity Index, PsARC = psoriatic arthritis response criteria, SSZ = sulfasalazine, ST = symptomatic therapy.

## 4. Discussion

### 4.1. Advances in understanding psoriatic arthritis pathophysiology

PsA is a complex immune-mediated disease involving multiple cytokine pathways, which has driven the development of targeted biological therapies. Traditional treatments, such as TNF inhibitors, marked the first wave of biologics for PsA, demonstrating significant efficacy in managing joint and skin symptoms.^[28,34]^ However, the limitations of TNF inhibitors, including loss of efficacy over time, partial responses, and adverse effects, have spurred research into alternative pathways involved in PsA pathogenesis. Advances in understanding the roles of interleukins, particularly IL-17 and IL-23, have led to the development of newer biological agents that offer improved specificity and efficacy.^[35]^

### 4.2. IL-17 inhibitors: expanding options for PsA treatment

IL-17 is a key cytokine in the inflammatory processes underlying PsA. The introduction of IL-17 inhibitors, such as secukinumab and ixekizumab, has significantly advanced PsA management by providing targeted suppression of this cytokine.^[31]^ Emerging therapies like bimekizumab, which inhibits both IL-17A and IL-17F, represent an evolution of this approach, offering enhanced efficacy compared to single-cytokine inhibitors.^[32]^ Clinical trials have shown that bimekizumab achieves robust improvements in joint inflammation, skin lesions, and difficult-to-treat manifestations such as enthesitis and dactylitis.^[24,25]^ These therapies have demonstrated high American College of Rheumatology (ACR) response rates and long-term safety, establishing their potential as first-line or second-line treatments for PsA.^[33]^

### 4.3. IL-23 inhibitors: precision targeting of PsA inflammation

The IL-23/IL-17 axis plays a central role in PsA pathogenesis. While earlier therapies, such as ustekinumab, targeted both IL-12 and IL-23, newer biologics like guselkumab and risankizumab provide selective inhibition of IL-23.^[34]^ This precision targeting minimizes off-target effects and offers superior control over PsA symptoms, particularly in patients who have failed TNF inhibitors.^[35]^ Guselkumab has shown consistent efficacy in achieving ACR20, ACR50, and ACR70 response rates, as well as significant improvement in skin psoriasis and nail disease.^[26,27]^ Risankizumab has also demonstrated encouraging results, with high remission rates and favorable safety profiles in clinical trials.^[36]^

### 4.4. Emerging pathways and novel targets

In addition to IL-17 and IL-23 inhibitors, research is exploring other pathways and targets implicated in PsA. Janus kinase (JAK) inhibitors, although not biologics, represent an adjacent therapeutic innovation that disrupts cytokine signaling upstream.^[37]^ New biological agents are also targeting cytokines such as IL-36 and granulocyte-macrophage colony-stimulating factor (GM-CSF), which may play roles in refractory PsA cases.^[38]^ While these therapies are in early stages of development, their novel mechanisms of action provide hope for addressing unmet needs in PsA treatment.

### 4.5. Efficacy and safety of emerging biologics

Emerging biologics have shown significant efficacy in treating both joint and skin manifestations of PsA. Clinical trials consistently report high ACR response rates, faster onset of symptom relief, and long-term maintenance of disease control. For instance, bimekizumab not only improves musculoskeletal symptoms but also offers superior control of skin psoriasis compared to earlier biologics.^[39]^ Similarly, guselkumab has been shown to effectively manage enthesitis and dactylitis, 2 of the most challenging features of PsA.^[27]^

Safety profiles of emerging biologics are also encouraging, with fewer systemic side effects compared to conventional treatments. Targeted therapies such as IL-23 inhibitors have demonstrated lower risks of infection and other adverse events, enhancing their suitability for long-term use. However, close monitoring remains essential, particularly for patients with comorbidities or those transitioning between therapies.^[40]^

### 4.6. Clinical implications and future directions

The introduction of emerging biologics has transformed the therapeutic landscape of PsA, enabling personalized treatment strategies based on disease phenotype and patient preferences. These therapies provide new options for patients who do not respond adequately to existing treatments, offering hope for improved outcomes and quality of life. The continued development of biomarkers to predict treatment response may further refine therapy selection, ensuring optimal use of these advanced agents.^[41]^

Looking forward, ongoing research into novel targets and combination therapies promises to expand the therapeutic arsenal for PsA. Advances in biologics, combined with innovations in small molecules and gene therapies, may eventually lead to more comprehensive disease control and even disease-modifying approaches. This dynamic field highlights the importance of continued investment in clinical trials and real-world studies to evaluate long-term efficacy, safety, and cost-effectiveness of emerging biologics.^[42]^

## 5. Conclusion

This study provides valuable insights into the efficacy and safety of various DMARDs in the treatment of PsA. Biologic DMARDs, particularly TNF inhibitors, IL-17 inhibitors, and IL-12/23 or IL-23 inhibitors, have proven to be more effective than conventional synthetic DMARDs in managing the symptoms of PsA, offering significant reductions in both joint and skin inflammation.^[43]^ While biologic treatments show high response rates and manageable safety profiles, some adverse effects such as infections remain a concern.^[44]^ Conventional treatments like cyclosporine and leflunomide were also effective, but with less pronounced benefits and a higher incidence of side effects.^[20]^ Targeted synthetic DMARDs, such as apremilast and deucravacitinib, have emerged as promising alternatives, demonstrating good safety and efficacy.^[45]^ These findings highlight the potential of biologic and targeted therapies in providing superior outcomes for PsA patients, emphasizing the importance of individualized treatment approaches to improve disease management and quality of life. Further research is needed to better understand the long-term benefits and risks of these treatments.

## Author contributions

**Conceptualization:** Saman Firdous, Muhammad Umair Choudhary, Muhammad Waqas Afzal, Rana Hamza Tayyab, Abida Parveen.

**Data curation:** Abida Parveen.

**Formal analysis:** Saman Firdous.

**Methodology:** Rana Hamza Tayyab.

**Project administration:** Muhammad Waqas Afzal.

**Resources:** Hamza Amjad, Muhammad Waqas Afzal, Abida Parveen.

**Supervision:** Saman Firdous.

**Validation:** Muhammad Umair Choudhary, Muhammad Waqas Afzal, Abida Parveen.

**Visualization:** Hamza Amjad, Muhammad Umair Choudhary, Rana Hamza Tayyab.

**Writing – original draft:** Hamza Amjad, Muhammad Umair Choudhary, Muhammad Waqas Afzal, Abida Parveen.

**Writing – review & editing:** Saman Firdous, Hamza Amjad, Muhammad Umair Choudhary, Muhammad Waqas Afzal, Rana Hamza Tayyab, Abida Parveen.
